# Genome-wide identification and functional dissection of the *BES1* family reveals key regulators of alkali stress response in hemp

**DOI:** 10.3389/fpls.2026.1819515

**Published:** 2026-04-30

**Authors:** Zeyu Jiang, Guochao Qi, Lina Wang, Ye Che, Di Wang

**Affiliations:** Institute of Hemp Science, Daqing Branch of Heilongjiang Academy of Agricultural Sciences, Daqing, China

**Keywords:** alkali, CsBES1 gene family, expression verification, HEMP, stress response, structural characterization

## Abstract

Plants frequently encounter diverse abiotic stresses, which require precise transcriptional regulation to maintain basic survival plasticity. The BES1 transcription factor family, a key component of brassinosteroid (BR) signaling, plays critical roles in plant development and stress responses. However, the genomic characterization, evolutionary characteristics, and biological functions of the BES1 family in industrial hemp (*Cannabis sativa* L.) remain unclear. In this study, we conducted a comprehensive genome-wide analysis of the *BES1*-like gene family in hemp and classified the 7 identified *CsBES1* genes into 5 branches based on their structural and phylogenetic association analysis. Gene promoter analysis revealed that cis-regulatory elements related to development, hormone signaling, and environmental stress (mainly BOX4, G-box, ARE, and ABRE) exhibited a dense distribution, indicating that they are involved in a complex regulatory network. Integrated RNA-sequencing data and gene expression profiling through quantitative real-time polymerase chain reaction (qRT-PCR) analysis showed that *CsBES1-2* and *CsBES1-3* genes had a good response towards alkaline stress. Subsequently, the subcellular localization of these two genes showed the cytoplasmic and nuclear distributions are indicative of a phosphorylation-dependent regulatory mechanism. The functional analysis through Yeast Two-Hybrid (Y2H) assays confirmed the activation of transcriptional activity of *CsBES1-2* and *CsBES1-3* genes and revealed enhanced alkali tolerance in yeast by showing the potential gene expression. In crux, this study provides the first systematic genomic characterization of the BES1 family in hemp and identifies *CsBES1-2* and *CsBES1-3* as key candidates for improving alkali stress resilience, offering valuable genetic resources for hemp breeding and crop improvement.

## Introduction

Industrial hemp (*Cannabis sativa* L.), which is known as a multipurpose commercial crop, has long been cultivated worldwide to satisfy anthropogenic activities for its tough fiber, oily grains, and medicinal secondary metabolites (Fike, 2016; Hazekamp and Fischedick, 2012; Viskovic et al., 2023). Soil is the most important part of growing crops because it gives plants the physical support and nutrients they need to grow and thrive in different conditions (Jiang et al., 2025). Over the past few decades, various salinity components have accumulated intensively on the soil surface due to soil erosion, evapotranspiration, and synthetic fertilization, making saline-alkali the most threatening abiotic stress to agricultural productivity and soil health. The Songnen Plain in northeast China, covering approximately 23,926 km², is characterized by a typical saline-alkaline landscape rich in sodium carbonate (Na_2_CO_3_) and sodium bicarbonate (NaHCO_3_) (Qiao et al., 2015), which adversely impacts the sustainable development of arable land and crop security (Sahab et al., 2021).

The previous research has reported a systemic destructive effect of alkali stress on hemp seedlings (Jiang et al., 2025). Phenotypic symptoms revealed that foliar dehydration-induced curling, followed by necrosis and extensive chlorosis, occurred obviously under stress induction. Histological observations further uncovered the ultrastructural damage involved with uneven thylakoid arrangement, reduced grana lamellae, and degraded starch granules, indicating severe impairment of photosynthetic assimilation under alkali stress (Jiang et al., 2025). Physiological analysis suggested that alkali stress triggered a significant oxidative stress to membranal lipid metabolism in markers of malondialdehyde (MDA) and reactive oxygen species (ROS) accumulation, indicative of the severe damage to the cell membrane integrity (Hu et al., 2019). Besides, declined morphological indices (e.g., plant height, fresh weight, and leaf number) also reflected the devastating mechanism of saline stress to the agronomic traits of hemp (Cafaro et al., 2025).

Brassinosteroids (BRs) are a class of plant-specific steroid hormones that play a fundamental role in regulating a broad range of plant growth and development, including promoting cell proliferation/elongation and division, enhancing crop resistance, and improving plant metabolism (Guo et al., 2013; Nawaz et al., 2017; Liu et al., 2024). Numerous signaling molecules are mutually regulated to generate a cascade network pathway in response to external stresses under stress-induced conditions. Up to the present time, the major BR signaling pathway has been extensively studied, commencing with BRI1 (membrane-localized receptor BRASSINOSTEROID INSENSITIVE 1) and BAK1 (co-receptor BRI1-ASSOCIATED RECEPTOR KINASE 1) and ending up with the activation of BES1 (BRI1-EMSSUPPRESSOR) and BZR1 (BRASSINAZOLE-RESISTANT 1) transcription factors. BES1 and its homolog BZR1 are the most significant effectors that trigger BR signaling in a positive way, which could be rapidly activated by PP2A (PROTEIN PHOSPHATASE 2A) to bind specific elements for enhancing BR pathway gene expression level and ultimately initiate BR-response events (Nolan et al., 2017; Tang et al., 2010; Tang et al., 2011; He et al., 2005).

BES1 and BZR1 belong to the *BES1* gene family, which ubiquitously exists in plants. The earlier reported research has determined approximately 88% similarity in properties in protein sequences of BES1 and BZR1 and 97% in N-terminal domains, as well as the fact that they collectively contain a bHLH domain at the N-terminus, forming a systematic response mechanism of these TFs to accurately bind to certain promoter regions of target genes, thereby regulating how plants cope with stress conditions (Salladini et al., 2020; Mao and Li, 2020). To date, more than 150 *BES1* genes have been discovered in multiple crops, e.g., *Arabidopsis thaliana*, *Brassica rapa* pekinensis (*Napa cabbage*), *Gossypium* (cotton), *Eucalyptus grandis* (flooded or rose gum), *Oryza sativa* (rice), and *Zea mays* (maize) (Cheng et al., 2023; Liao et al., 2020), and the function of 30 BES1 transcription factors (TFs) has been clearly classified in plant growth, developmental, and stress responses (Liu et al., 2018). For instance, it was reported that plants can keep a normal growth state of roots under phosphorus deficiency phases through continuous biosynthesis of BES1/BZR1 (Pandey et al., 2020). It was also reported that the transcriptional level of BES1 positively induced the responses of *Arabidopsis thaliana*, *Brassica napus* (rapeseed), and *Oryza sativa* (rice) to abiotic stresses, including drought (insufficient water), heat (excessive temperature), and cold (low-temperature) stress (Kitazumi et al., 2018; Song et al., 2018; Chen et al., 2019).

Brassinosteroid (BR) signaling, mediated by the *BES1* gene family of transcription factors, is a cornerstone of plant adaptation to abiotic stresses, particularly salinity and alkalinity. To date, most effort in searching the function of the BES1 gene family is limited in *Arabidopsis thaliana* and alike model plants. Thus, it highlights the significance of extending BES1-related research in other crops, such as hemp with economic value for providing abundant industrial materials. Herein, a series of bioinformatic techniques were utilized to analyze the physicochemical properties, conserved domains, and expression patterns of the *CsBES1* gene family in hemp for the purpose of demonstrating the following: (1) the number and physicochemical properties of the *CsBES1* gene family members in industrial hemp; (2) the analysis of the conserved domains, phylogenetic tree, conserved motifs, and cis-acting elements of the *CsBES1* gene family members; and (3) the tissue-specific expression profiles in different organs and the screening for key candidate genes of the *CsBES1* gene family in hemp to regulate alkali stress. The obtained results would provide a comprehensive perspective for understanding the *BES1* gene family characteristics and the specific gene function in hemp under alkali stress.

## Materials and methods

### Research material

The industrial hemp variety [Qingma 1 (QM1)] was selected as the research material due to its alkaline susceptibility, as shown in our previous study (Jiang et al., 2025). In brief, the seed of this variety was offered by the Institute of Hemp, Heilongjiang Academy of Agricultural Sciences (HLJAAS), Daqing Branch, China. Alkali-induced stress treatment (250 mM stress of sodium bicarbonate (NaHCO_3_)) and control treatment (CK, distilled water (ddH_2_O), with no stress) were applied in a completely randomized design (CRD) using three replications consisting of 3 uniform hemp seedlings; the alkali-stressed and CK groups were randomly arranged in the controlled environmental conditions, including temperature (25 °C day/20 °C night) and photoperiod (16 hr light/8 hr dark) in an artificial chamber to avoid positional effects. Concerning the rationale for selecting the 250 mM NaHCO_3_ concentration, we conducted a preliminary series of lab experiments to screen appropriate stress concentrations, and 250 mM was selected as the finalized treatment.

For the seedling cultivation, seeds were soaked in muslin bags for two days at a temperature of 30 °C. Later, all the sprouted seeds were shifted into a soil-grown system containing organic carbon (OC) = 20% (weight percentage (WP, %) on dry product), organic nitrogen (ON) = 1%, and organic matter (OM) = 35%. The pH of the CK and alkali-treated soil system was measured with a digital pH meter (LB-TPH, Loobo, Tsingtao): for CK and alkali-stressed groups, pHs ranged between 6.68 and 7.12 and 8.87 and 9.38, respectively. Besides, the plants were grown under optimal environmental conditions with a day/night temperature regime of 25 °C/20 °C, a photoperiod of 16 h light/8 h dark, a light intensity of 12,000 lux, and a relative humidity of 60 ± 5.0%. The alkali-induced stress and control treatments were applied when the seedlings arrived at the four-leaf stage (about 8-12 cm). The samples from different developmental organs [seeds, roots, stalks, and leaves (without stress treatment)] were collected for tissue-specific gene expression validation; however, the alkali-treated samples and CK groups from dissimilar time laps (0 h, 6 h, 24 h, and 48 h) were collected for determining stress-induced gene profiles.

### Detection of *CsBES1*-like gene family members by bioinformatics approach

The genome-wide bioinformatics analysis was conducted based on the earlier reported studies on gene family analysis (Yang et al., 2024; Zhang et al., 2023). The whole-genome database of hemp (Finola) was obtained from http://gdb.supercann.net/ (accessed on 5 September 2025). The protein sequences of BES1/BZR1 of Arabidopsis were copied from the Arabidopsis Information Resource (TAIR database, http://www.arabidopsis.org/, accessed on 10 September 2025), which were used as a batch query to search through the hemp genome by the BLASTP package with the expectation (E) value < 1e-5 to obtain all identified candidate genes that may have the BES1/BZR1 domain by the web-based resource of Simple Modular Architecture Research Tool (SMART) databases (http://smart.embl-heidelberg.de) that identify annotated protein domains and their architecture. Additionally, the differential physical and chemical attributes of BES1/BZR1-like protein sequences were analyzed using the prominent bioinformatics tool “Protparam” (https://web.expasy.org/protparam/); however, the subcellular localization(s) and the associated membrane type(s) were predicted by the multi-label predictor “DeepLoc” (version 2.1, https://services.healthtech.dtu.dk/services/DeepLoc-2.1/). Ultimately, based on the hemp genomic information, TBtools-II (upgraded version) was utilized to exhibit gene distribution around the chromosomes (Chen et al., 2023).

### Phylogenetic association of the BES1 family

The multiple amino acid sequences from hemp, soybean (*Glycine max*), Arabidopsis (Arabidopsis thaliana), and tomato (*Solanum lycopersicum*) were retrieved through the Soybean Genome Annotation Project database (https://phytozome.jgi.doe.gov/pz/portal.html, accessed on 15 September 2025), the Arabidopsis Information Resource (TAIR) database (https://www.arabidopsis.org/), and the Solanaceae Genomics Database (http://solgenomics.net/, Tomato Genome proteins (ITAG release 4.0), accessed on 20 September 2025) were obtained jointly to conduct multiple sequence alignments using ClustalX (version 2.10). The alignments were then used for analyzing the phylogenetic association by the likelihood neighbor-joining (NJ) method with a bootstrap value >1000 in MEGA XII (Kumar et al., 2024).

### Conserved motif and domain identification

The structure of *BES1*/*BZR1* genes was graphically visualized by TBtools-II (upgraded version) (Chen et al., 2023), and the related conserved protein motifs were searched via Multiple Em for Motif Elicitation “MEME” Suite (version 5.5.9, http://meme-suite.org/tools/meme, accessed on 25 September 2025) with the forecast quantity of 10 (Yang et al., 2024; Zhang et al., 2023). Additionally, the Batch CD-Search from NCBI was utilized to identify the overall conserved domains of BES1/BZR1 family members.

### Tissue-specific gene expression validation under stress

The real-time quantitative polymerase chain reaction (RT-qPCR) was performed to accurately measure the genetic expression profiles of CsBES1 family genes, following a previously described method (Brands and Ho, 2002). The high-quality total RNA from collected plant tissues was extracted using the RNAprep Pure Plant Plus Kit (DP441) from TIANGEN Biotech, China. The integrity and concentration of RNA samples were checked by agarose gel electrophoresis and spectrophotometry. First-strand complementary/copy DNA (cDNA) was synthesized from 1 μg of high-quality purified RNA using the TransScript^®^-Uni One-Step gDNA Removal and cDNA Synthesis SuperMix kit [(AT311), TransGen Biotech, Beijing, China], based on the practical guidance manual of the manufacturer (Bustin et al., 2009). The specificity of the designed primers was verified by melting curve analysis, and only a single peak was observed. Amplification efficiency was estimated using a 5-fold serial dilution of cDNA template, and values ranging from 90% to 110% were accepted for further analysis.

The gene-specific primers were generated by manually operating the Primer Premier (version 6.10) software, and exported primer sequences (forward and reverse) were oligo-synthesized by Sangon Biotech (Shanghai, China). RT-qPCR reactions were carried out using SYBR Green chemistry on a real-time PCR detection system under standard cycling conditions. The housekeeping gene *CsActin* was used as an internal reference to normalize gene expression levels (Czechowski et al., 2005). The gene expression patterns were evaluated based on the 2^−ΔΔCt^ method (Livak et al., 2001) by means of three biological repetitions for each sample collected at different time courses. The detailed information of the exported gene primer is exhibited in **Table 1**. Further, we used our own RNA-sequencing data, uploaded and publicly available on the NCBI website, under BioProject (Accession: PRJNA1163756). The detailed statistics of reads mapping of RNA-seq data are available in the supplementary table of our previously published study (Jiang et al., 2025).

**Table 1:** The information of the identified *CsBES1* gene primers used for qRT-PCR analysis.

Gene name	Gene ID	Forward primer (sense)	Reverse primer (antisense)
*CsBES1-1*	*LOC115706510*	CAGCCTCAGGGTTCAAGACAT	ATAGTTGAAGTTTGAGAACGAGCAC
*CsBES1-2*	*LOC115706839*	CTCATCGGCATCCTCATCTG	GACGCCCTGGACTTGGTG
*CsBES1-3*	*LOC115714441*	ACCGAAAGGGAACTCAGCC	AGATAATTGGAGGGGTTTGCAT
*CsBES1-4*	*LOC115697246*	TTAGATTCTCAACCACCTTCGG	AATCGTTCCCATTATTTGTAGAGC
*CsBES1-5*	*LOC115697642*	CTGGCTGGACCGTTGAGG	TACGTTGGCAGTGTAATGAGATG
*CsBES1-6*	*LOC115698180*	ATTCCGATGGCGCTTTATGA	CGAGACTCCAACAATGGGTGA
*CsBES1-7*	*LOC115700469*	GGAGGCGAAGAGCCATAGC	GGGAAGCGTAAGGGGTGATT
*Actin*		CCAATAGCCTTGCATTCCAT	TCGATTGGAAAGCCGAATAC

### Subcellular localization of *CsBES1* proteins

Subcellular localization analysis was conducted by fusing the full-length coding sequence (CDS) of the target *CsBES1* genes into the specialized binary plant expression vector (pBI121-EGFP) derived from the pBI121 plasmid. To obtain the high transformation efficiency, the recombinant constructs were introduced into the GV3101 Agrobacterium tumefaciens strain (GoldBio’s) and subsequently infiltrated into the eight-leaf stage of tobacco (*Nicotiana benthamiana*). After 3 days of incubation, GFP fluorescence signals were observed using a high-performance, fully automated laser scanning confocal microscope (LSCM) with a white light laser (WLL) (Leica TCS SP8, Germany), following previously described methods (Sparkes et al., 2006; Goodin et al., 2008).

### Transactivation activity and function identification in yeast

The open reading frames (ORFs) of the *CsBES1* genes were cloned into the pGBKT7-GAL4BD vector (that expresses proteins fused to amino acids) and subsequently transformed into the Yeast Two-Hybrid (Y2H) Gold yeast strain system. Transformants were screened on three types of selective synthetic dropout (SD) media: SD/–Trp, SD/–Trp–His supplemented with X-α-gal, and SD/–Trp–His–Ade supplemented with X-α-gal. The transcriptional activation activity of the fused proteins was assessed by α-galactosidase activity, indicated by blue coloration on X-α-gal–containing media (Fields and Song, 1989; Clontech Laboratories, Inc., 2013).

For yeast stress tolerance assays, the ORFs of the *CsBES1* genes were amplified and inserted into the restriction sites of endonucleases (*Hind*III and *Xba*I) of the yeast expression vector (pYES2-NTB). The constructs of the recombinant and the empty vector of pYES2-NTB were induced into the yeast strain (INVSc1) using the lithium acetate-mediated transformation method according to the manufacturer’s instructions (Invitrogen, Carlsbad, CA, USA) (Gietz and Schiestl, 2007). Transformed yeast cells were cultured in YPDA liquid medium at 30 °C overnight. The prepared cultures were then amended to an OD_600_ of 0.6 and shifted to SG–Ura induction medium supplemented with 2% (w/v) galactose for 24 h to induce *CsBES1* expression. Subsequently, 3.0 μL of serially diluted yeast suspensions (10^0^, 10^−1^, and 10^−2^) were spotted onto SG-Ura solid medium supplemented with 0, 10, or 15 mM NaHCO_3_ and incubated for 7 days to assess yeast growth under alkali stress conditions (Invitrogen, 2010; Zhang et al., 2016).

### Statistical data analysis

The experimental data were recorded as numerical values and initially processed by Microsoft Excel Worksheet (Microsoft Corporation, 2021). The finalized datasets were subsequently evaluated for statistical differences and graphically represented by operating GraphPad Prism software (version 10.0) (GraphPad Software, Inc., 2023). Statistical analyses were conducted at significance levels (p < 0.05 and p < 0.01).

## Results

### Analysis of chromosomal distribution of *BES1*-like gene family members

The protein sequence of *Arabidopsis thaliana* (AtBES1/AtBZR1) was used as the query; we identified seven *CsBES1*-like genes in the hemp genome, designated *CsBES1-1* to *CsBES1-7*, all of which contain the characteristic BES1_N domain. Regarding the genomic distribution, the seven *CsBES1* genes are unevenly mapped across four chromosomes: two on chr01 (*CsBES1-1* and *-2*), one on chr04 (*CsBES1-3*), three on chr07 (*CsBES1-4, -5, -6*), and one on chr10 (*CsBES1-7*), whereas *CsBES1-3* and *CsBES1-7* are located individually on chr04 and chr10, respectively. This non-random distribution pattern, particularly the aggregation on chr01 and chr07, suggests that segmental or tandem duplication events have played a pivotal role in the lineage-specific expansion of the *CsBES1* family in hemp.

**Figure 1:**
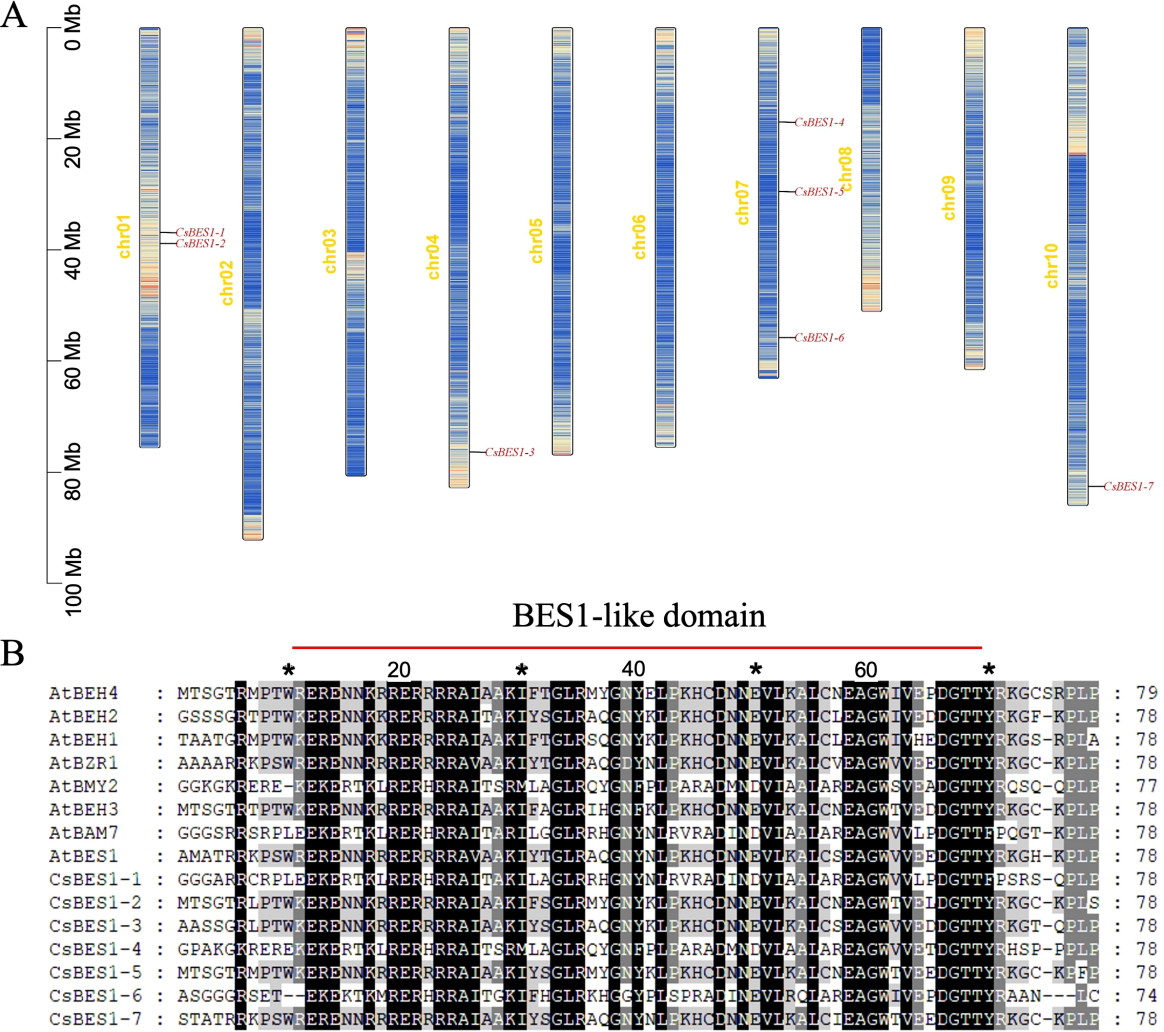
Analysis of the *CsBES1* gene and protein component distribution within the hemp genome. **(A)** Localization of the *CsBES1* genes across the chromosomes; **(B)** Comparative sequence alignment of the BES1-like domain in the proteins of hemp and Arabidopsis.

The physicochemical analysis highlighted the diversity and conservation within the CsBES1 family. While the full-length proteins exhibit significant variation in amino acid length (207-720 aa), molecular weight (21.6-81.2 kDa), and isoelectric point (pI 5.59-9.14, with five basic and two acidic members) (**Table 2**), the core functional architecture remains strictly preserved. Comparative sequence analysis confirmed that the BES1_N DNA-binding domain is highly conserved across all members, ranging from 115 to 173 aa (**Figure 1B**). This structural integrity exhibited the strict functional constraints on the DNA-binding interface, thereby ensuring the preservation of transcriptional regulatory capacity (Liu et al., 2018). Consistently, subcellular localization predictions indicate that all seven *CsBES1* proteins are localized to the nucleus. Taken together, the conserved family size, the intact DNA-binding domain, and the uniform nuclear localization indicated that the *CsBES1* gene family has undergone intense purifying selection during evolution to ensure that its core function as a transcription factor in regulating growth and development remains highly conserved and irreplaceable.

**Table 2:** The chromosomal distribution and physicochemical attributes of *CsBES1*-like genes in hemp.

Name of gene	ID of gene	Chromosomal position	Length/aa	MW/Da	pI	Localization
*CsBES1-1*	*LOC115706510*	chr01:36840201-36846857	704	79028.78	5.74	Nucleus
*CsBES1-2*	*LOC115706839*	chr01:38814462-38817886	325	34665.76	8.74	Nucleus
*CsBES1-3*	*LOC115714441*	chr04:76362107-76364638	320	34253.28	8.99	Nucleus
*CsBES1-4*	*LOC115697246*	chr07:16972006-16989599	720	81195.23	5.59	Nucleus
*CsBES1-5*	*LOC115697642*	chr07:29503033-29508025	322	34850.64	8.08	Nucleus
*CsBES1-6*	*LOC115698180*	chr07:55755102-55756540	207	21656.1	8.40	Nucleus
*CsBES1-7*	*LOC115700469*	chr10:82533668-82535584	317	34300.43	9.14	Nucleus

Molecular weight (MW), Dalton (Da), amino acid (aa), and isoelectric point (pI).

### Analysis of conserved motifs and domains

**Figure 2:**
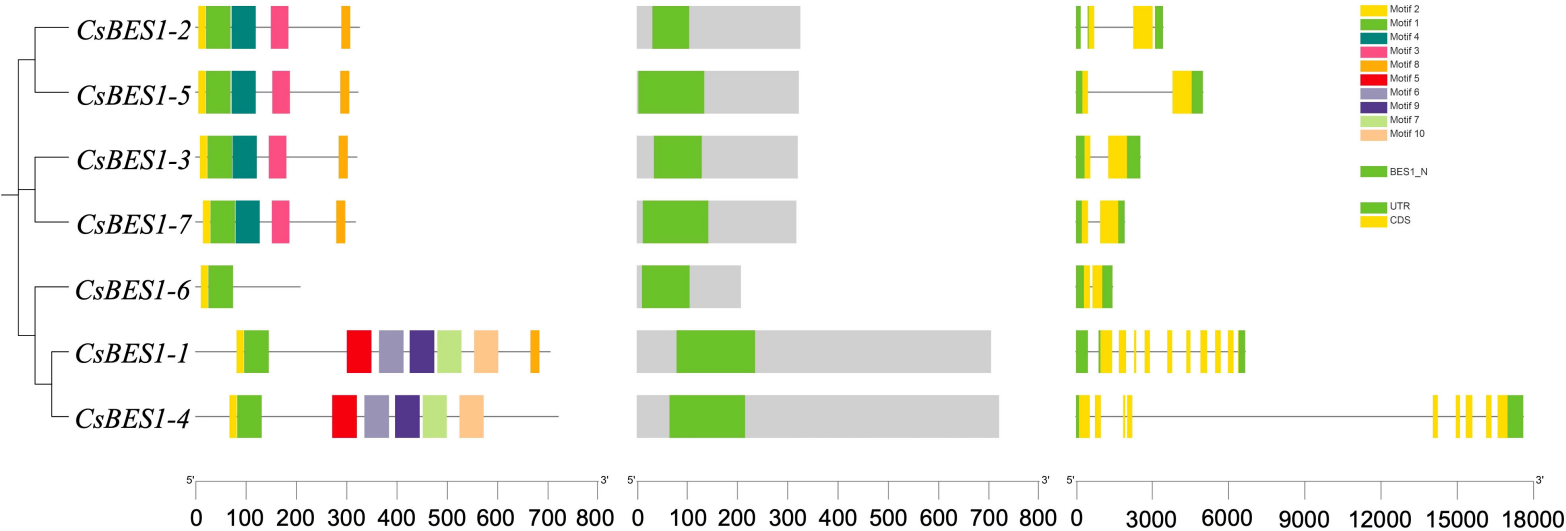
The conserved motifs and structures of identified candidate *CsBES1* genes, respectively.

To elucidate the structural and functional divergence of the *CsBES1* family, we analyzed the distribution of conserved motifs and exon-intron architectures (**Figure 2**). Within the identified 10 conserved motifs, motifs 1, 2, and 8 were universally present across all seven *CsBES1* members. This high conservation of the BES1_N DNA-binding domain maintained the stability of core transcriptional regulatory function. Besides, a distinguished structural feature was also observed among phylogenetic subgroups, reflecting functional specialization. For example, *CsBES1-2, -3, -5*, and *-7* exhibited a compact genomic structure with only 2 exons and 1-3 introns, while *CsBES1-1* and *-4* displayed a more complex architecture, containing 9 exons and 8-10 introns. Such pronounced variation in gene structure is not merely random but likely results from specific evolutionary mechanisms driving family diversification.

### Analysis of phylogeny of *BES1*-like genes

**Figure 3:**
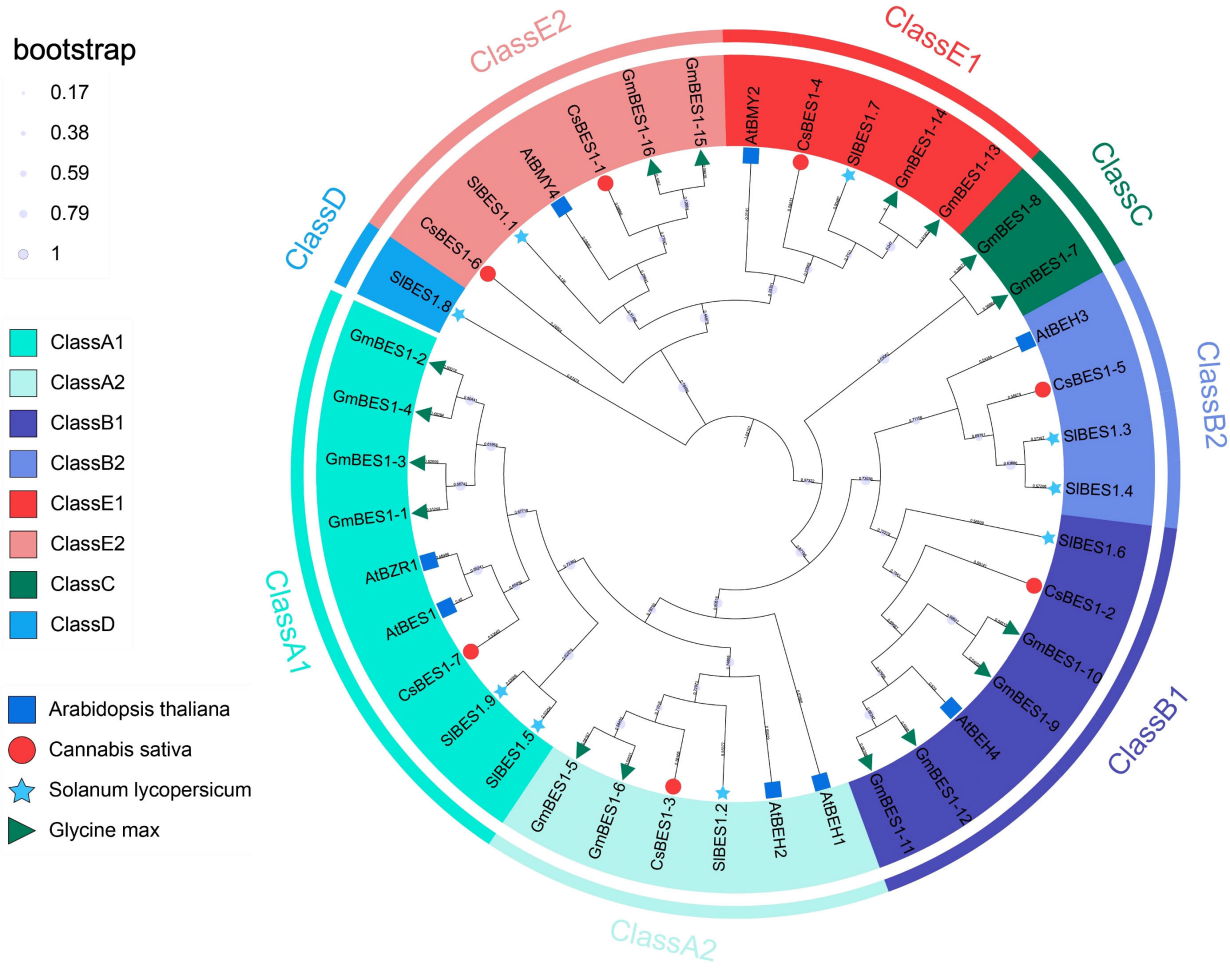
Phylogenetic association analysis of the *CsBES1*-like gene family across hemp, Arabidopsis, tomato, and soybean based on the neighbor-joining method. Different colors are representing the clustered groups and subgroups among the classes, respectively.

To infer the evolutionary relationships and functional divergence of the *CsBES1* gene family, we constructed a maximum-likelihood phylogenetic tree using full-length protein sequences from *C. sativa*, *A. thaliana*, *S. lycopersicum*, and *G. max* (**Figure 3**), with all the identified 40 BES1-like proteins clustered into five distinct clades (Classes A-E). It is noticeable that the majority of genes (>90%), including all canonical BES1 and BZR1 members, are clustered into Class A, B, and E. Within these conserved clades, *CsBES1-2* and *CsBES1-3* grouped tightly with Arabidopsis *AtBES1* and *AtBZR1*, respectively, identifying them as the likely functional orthologs in hemp. In contrast, Class C and D exhibited striking species-specific distribution patterns. Class C contained exclusively soybean genes (GmBES1-7, 8), while Class D was represented solely by a tomato member (SlBES1.8).

### Analysis of cis-element structure of *CsBES1*-like genes

**Figure 4:**
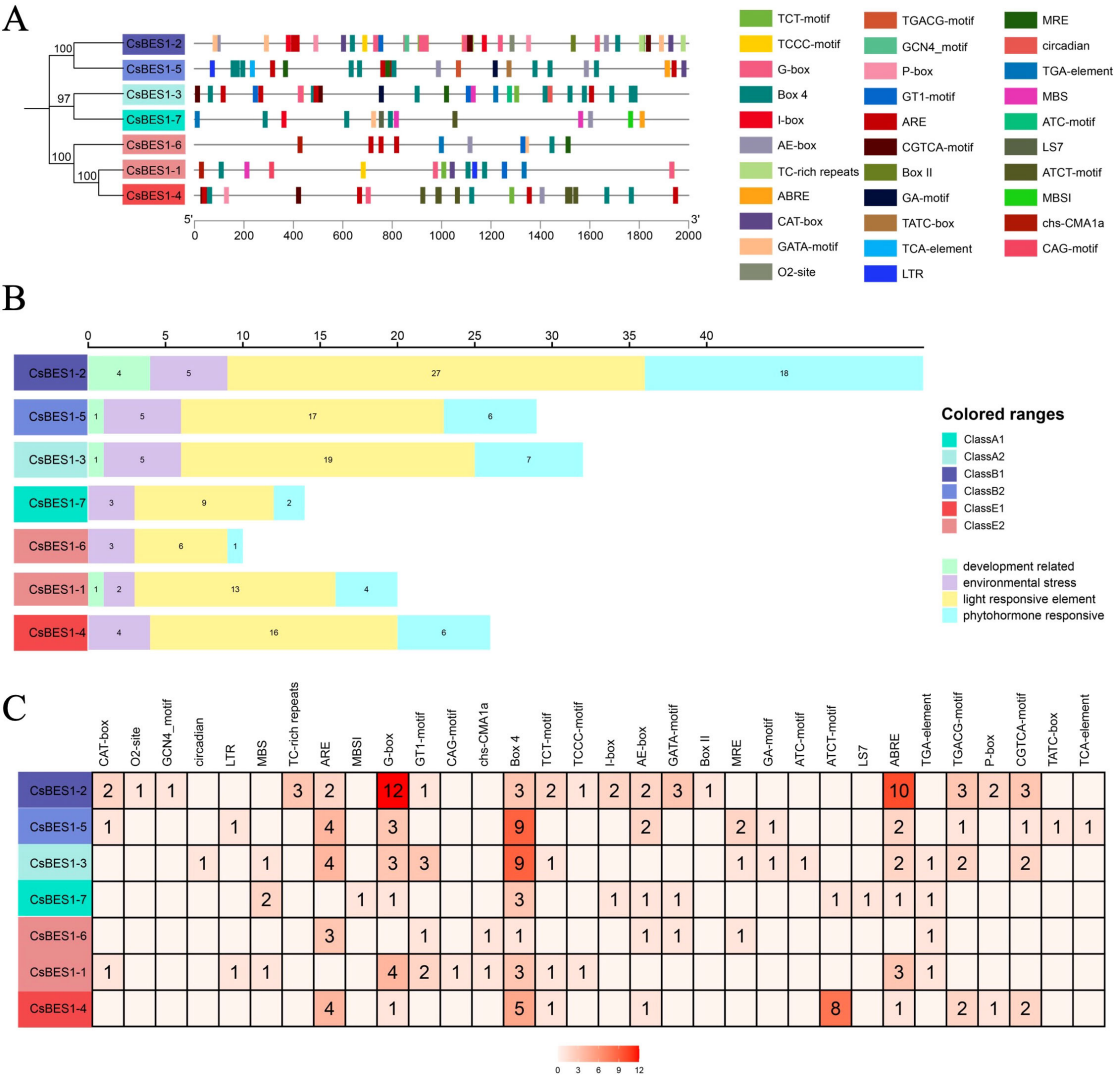
Structural analysis of cis-acting elements of *CsBES1*-like genes in hemp. **(A)** Locations of cis-elements in *CsBES1* genes. **(B)** The recapitulative clusters of the elements distributed in *CsBES1* genes. **(C)** The detailed information of the identified cis-elements in *CsBES1* genes. Different colored blocks are representing the distinguished cis-acting elements, respectively.

To explore the distinguished or synergistic cis-element regulation mechanism in hemp, DNA sequences having 2000 bp from upstream of *CsBES1*-like genes were deposited into the database of PlantCARE after removal of the basic and unidentified elements. In total, 32 cis-elements authenticated from the database have been clustered into four types: development-related, environmental stress, light-responsive elements, and phytohormone-responsive elements (**Figure 4A**). BOX 4, G-box, ABRE, and ARE were remarkable as compared with the relative average distributed elements, which participate in regulating organic acid, primary metabolism, and adversity response, implying that the *CsBES1*-like genes may be induced by environmental alterations and help to enhance the stress resistance of hemp. Furthermore, *CsBES1-2, CsBES1-3, CsBES1-4*, and *CsBES1-5* possessed more complicated cis-element structures than the others, which highlights the essential role of these genes in operating independently or synergistically to improve the adaptive capacity of hemp (**Figures 4B, C**).

### Analysis for validation of tissue-specific and stress-induced *CsBES1* genes expression

**Figure 5:**
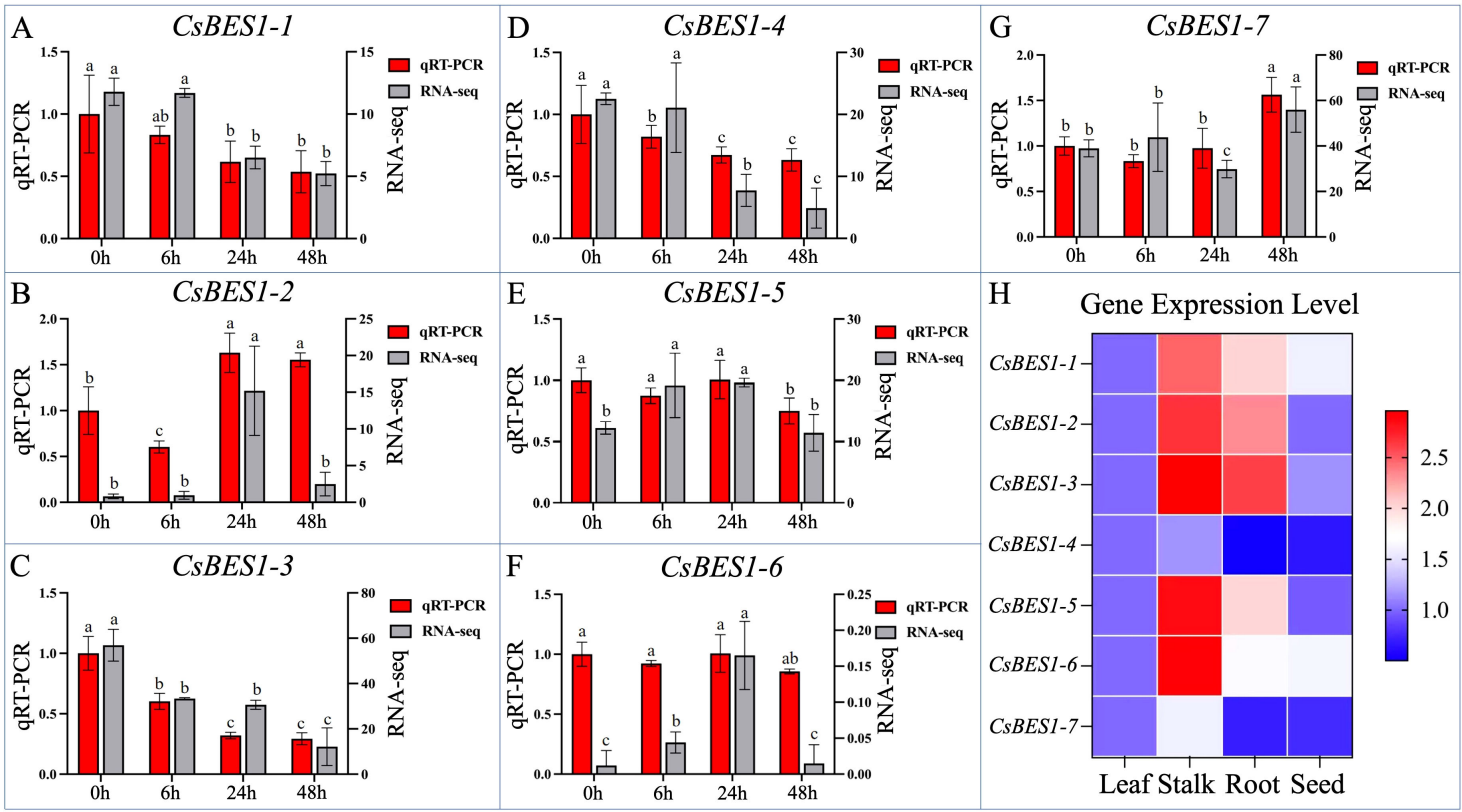
Transcriptomic validation and identified candidate gene expression patterns analysis in hemp. **(A–G)** show *CsBES1* gene expression in hemp subjected to alkali stress at various time intervals. Statistical letters (a, b, c) indicate significant differences at p < 0.05. **(H)** presents a heat map illustrating the tissue-specific *CsBES1* gene expression in hemp organs under alkali stress.

For the purpose of exploring the operating position of *CsBES1* genes, real-time quantitative polymerase chain reaction (RT-qPCR) was conducted to exhibit the tissue-specific validation pattern of all 7 *CsBES1* genes in hemp (**Figure 5**). In total, 12 samples from root, stalk, leaf, and seed, including three biological replicates, were collected and used for expression profiles of the key *CsBES1* genes. As shown by the bar graphs and heatmap, the *CsBES1* genes are expressed ubiquitously in the main organs of the stalk and root, which exhibited more drastic expression levels compared to the leaf and seed of hemp, implying a potential mechanism of BES1 genes in regulating stalk development and root architecture. It was noticed that the *CsBES1-2, CsBES1-3, CsBES1-5*, and *CsBES1-6* had the relatively higher expression pattern, which may fundamentally play the core role of the BES1 family to improve plant growth.

Considering the above-mentioned results indicating that the *CsBES1* genes showed a relatively drastic expression trend in the stalk, samples from different time intervals under alkali stress were collected to further analyze the response mechanism as well as the potential function of the *CsBES1* genes examined by RT-qPCR and transcriptomic (RNA-seq) data analysis. The overall *CsBES1* genes were expressed by the alkali stress and exhibited a downregulated trend in different degrees consistent with the previously reported alteration trend of BES1-like genes exposed to abiotic stresses. Among these, the hyperresponsive *CsBES1-3* under alkali stress decreased by 70.67% at 48 h compared with CK compared with the relatively mildly expressed *CsBES1-4, CsBES1-5*, and *CsBES1-6*, implying its passively regulated role in regulating hemp alkali tolerance. Nevertheless, the contrasting expression of *CsBES1-2* likely indicates its positive role in enhancing hemp’s resilience to adversity, with increases of 63.00% and 55.33% observed at 24 and 48 hours under alkali stress.

### Analysis of subcellular localization of CsBES1 proteins

To further verify the function position of CsBES1 proteins, *CsBES1-2* and *CsBES1-3*, which exhibit distinct expression profiles, were selected for subcellular localization analysis (**Supplementary Figure 1**). The *CsBES1*-GFP fusion constructs were transiently expressed in leaves of tobacco (*Nicotiana benthamiana*) via Agrobacterium-mediated infiltration. Fluorescent signals for both *CsBES1-2*-GFP and *CsBES1-3*-GFP were clearly detected in both the nucleus and the cytoplasm. This dual localization pattern is highly consistent with the established regulatory mechanism of BES1 family members, where phosphorylated BES1 predominantly resides in the cytoplasm, while dephosphorylated BES1 accumulates in the nucleus to function as a transcription factor (Yin et al., 2005). The presence of fluorescence in both compartments suggests that CsBES1 proteins undergo dynamic nucleocytoplasmic shuttling and likely act as transcriptional regulators that are actively transported into the nucleus to modulate downstream gene expression.

### Analysis of transactivation activity of CsBES1 proteins

Since the conserved domain characteristic of CsBES1-2 and 3 was identified as TFs, a yeast-based GAL4-responsive reporter system was subsequently constructed to determine the transactivation activity of CsBES1 members (**Supplementary Figure 2**). As shown, all groups had good growth status on SD-T medium, indicating that the plasmids were successfully transferred into yeast cells. As compared with the positive control PGBKT7-VP16 (a strong transcriptional activator), which exhibited blue fluorescence on SD-TH+X (Site-Directed/Structural Domain-Thiol-Modified + X) and SD-THA+X (Structural Domain-Thiol-Allocated/Modified + X) plates due to the transcriptional activation property of VP16, the negative control pGBKT7 cannot grow on both plates of SD-TH+X and SD-THA+X, indicative of the availability of the experimental system. The observed vigorous growth status of pGBKT7-*CsBES1-2, 3*, as well as pGBKT7-VP16-*CsBES1-2, 3*, in SD-TH+X and SD-THA+X both exhibited blue fluorescence, which was related to the pGBKT7-VP16 (positive control), revealing that *CsBES1-2, 3*, possess transactivation activity.

### Analysis of candidate *CsBES1* genes function verified by yeast system

To explore the function of *CsBES1-2* and *CsBES1-3* in alkali stress tolerance, we conducted yeast complementation assays under gradient alkali conditions (**Supplementary Figure 3**). Under normal conditions, all yeast strains showed similar growth. With increasing alkali concentration, the control strain (empty pYES2-NTB) exhibited growth inhibition, and its viability dropped sharply at concentrations ≥12 mM. In contrast, yeast expressing *CsBES1*-*2* or *CsBES1-3* displayed significantly improved growth, especially at 15 mM alkali stress, where the control strain barely grew. Although CsBES1 overexpression enhanced alkali tolerance, it did not confer complete resistance; cell viability decreased under extreme stress (≥20-50 mM), indicating the protective effect is limited and acts within a specific physiological range to boost basal stress resilience. Collectively, *CsBES1-2* and *CsBES1-3* are positive regulators of alkali tolerance. Their strong performance under moderate-to-high stress makes them promising candidates for hemp breeding. Introducing these genes into elite cultivars through molecular breeding or genetic engineering may improve the adaptability of hemp to saline-alkali soils, facilitating cultivation in marginal lands and stabilizing yields.

## Discussion

Hemp is a sustainable industrial crop based on its natural properties like minimal water and fertilizer needs, adaptive growth status, and pliable fiber, making it an ideal eco-repair agent as well as an economical raw material supplier. However, frequent climate change along with extreme soil salinization negatively threatened hemp yield, which elevated the necessity for saline-alkali resistance breeding of hemp (Jiang et al., 2025).

Genome-wide characterization of the BES1 family has been extensively documented in various crops, including Brassicaceae (rapeseed, mustard, and Chinese cabbage), monocots (maize), and legumes, collectively demonstrating expansion and functional diversification among different species (Saha et al., 2015; Song et al., 2018; Manoli et al., 2018; Li et al., 2018; Wu et al., 2016). In our study, the identification of *CsBES1*-like genes across distinct chromosomes (**Figure 1**) showed that this family size is comparable to that observed in *Arabidopsis* (8 members) and tomato (*Solanum lycopersicum*, 9 members) (Su et al., 2021). This family size is comparable to that observed in *Arabidopsis* (8 members) and tomato (*Solanum lycopersicum*, 9 members) (Su et al., 2021). Despite the distinct genome duplication events and divergent evolutionary trajectories experienced by these species, the conservation of the *BES1* family member count suggests strong functional constraints. This implies that the *BES1* signaling module has been strictly maintained throughout evolution, likely due to its indispensable role in brassinosteroids-mediated growth and development. In contrast, our comprehensive analysis reveals a unique evolutionary process characterized by significant structural conservation in hemp (**Figure 2** and **Figure 3**). The previous research demonstrated that insertion/deletion, exonization/pseudoexonization, and gain/loss mechanisms comprehensively regulate the exon/intron diversification among gene families (Xu et al., 2012). Consequently, amino acid substitutions and structural rearrangements have led to functional diversification, which suggested that, although the DNA-binding core remains conserved, the non-coding regions and auxiliary domains have undergone dynamic rearrangements, allowing hemp to adapt to different developmental stages and environmental conditions. The absence of hemp or Arabidopsis genes in these defined clades suggests lineage-specific expansion events in legumes, which is expectable because soybean is a palaeo-tetraploid plant harboring most genes in multiple copies due to the two WGD events (Schmutz et al., 2010).

Unlike the extensive gene duplication events observed in polyploid species, e.g., rapeseed, the *CsBES1* family in cannabis remains compact yet highly conserved, with all seven members retaining the same domain architecture and motif arrangement (**Figure 4**). This evolutionary stability suggests that these genes are subject to strict selective constraints. We propose that this compositional fixation reflects a critical constraint: the current *CsBES1* landscape in cannabis is highly stable and functionally robust, with functions likely essential for fiber development and basic stress tolerance, leaving little room for variation. Similarly, findings in Arabidopsis and cotton further indicate that the molecular mechanism governing BR signaling in hemp is an ancient and well-preserved module (Zhu et al., 2023). Therefore, this evolutionary perspective provides a crucial context for interpreting our subsequent functional data: any perturbation in these highly conserved *CsBES1* genes could have cascade effects on hemp’s growth and stress adaptability, underscoring their potential as precise targets for genetic improvement.

The traditional BR signaling cascade, wherein dephosphorylated BES1/BZR1 accumulates in the nucleus to activate target genes, is tightly modulated by post-translational modifications (PTMs) in response to environmental changes (Lv and Li, 2020). While the core phosphorylation/dephosphorylation switch mediated by BRASSINOSTEROID INSENSITIVE 2 (BIN2) and PROTEIN PHOSPHATASE 2A (PP2A) is well-established (Yin et al., 2002; Tang et al., 2011; Lv and Li, 2020), emerging evidence highlights SUMOylation as a critical rheostat for regulating BES1 stability and localization under stress. Specifically, salt stress has been shown to trigger deSUMOylation of BES1/BZR1, promoting their interaction with SUMO proteases (e.g., ULP1a) and facilitating their exclusion from the nucleus or destabilization, thereby rapidly shutting down growth-promoting signals (Srivastava et al., 2020). Herein, the subcellular localization data aligns with this dynamic regulatory model. The *CsBES1-2* and *CsBES1-3* proteins are distributed in both the nucleus and cytoplasm under normal conditions, but the alkali stress-induced transcriptional downregulation suggests a dual-layer suppression mechanism. We hypothesize that in hemp, alkali stress not only represses CsBES1 transcription but also likely induces deSUMOylation-mediated nuclear export of the existing protein pool. The observed nucleo-cytoplasmic distribution suggests that CsBES1 proteins are actively moving between these two areas, where the regulation towards cytoplasmic retention under stress aligns with the deSUMOylation effects reported in Arabidopsis and tomato (Jiang et al., 2015). This implies that hemp employs a conserved, rapid-response strategy by actuating transcriptional silencing of BES1, preventing BR-mediated growth to prioritize stress adaptation.

Overall, the BR signaling transduction pathway is mainly governed by the spatiotemporal dynamics of kinase/phosphatase activities in the cytoplasm and subsequent transcriptional regulation in the nucleus, collectively modulating plant growth, development, and stress responses. Central to this regulation are cis-acting elements in gene promoters, which mediate the specific binding of TFs to fine-tune gene expression in response to endogenous and environmental factors. As an element with multiple functions, the G-box was characterized as a light-responsive element in the ribulose 1,5-bisphosphate carboxylase/oxygenase (Rubisco) promoter, as well as implicated in regulating phytohormone-responsive genes (e.g., ethylene- and jasmonate-induced genes in tobacco) and regulating ABA-mediated stress resistance by binding *HVA22* in yeast (Meller and Fluhr, 1995; Xu and Timko, 2004; Shen and Ho, 1995). In this study, the 32 identified dominant cis-elements within the CsBES1-like promoters were clustered into four functional categories (**Figure 4**), 24 of which were G-box motifs, indicating their predominant regulatory feature of the CsBES1 family. Additionally, the non-random, high-density properties of G-boxes represent a species-specific evolutionary adaptation in hemp, which leads us to speculate that *CsBES1* is the integration hub tightly coupled with both BR signals and stress signals and is intensively regulated by ABA signals to integrate light perception and phytohormones in order to optimize stress resilience. Especially for *CsBES1-2* and *CsBES1-3*, the complicated and broad-spectrum features of their cis-element distribution further demonstrate their significant importance in regulating the BR-mediated signaling pathway.

While the pivotal role of ABA in regulating organ senescence, stomatal dynamics, and stress responsiveness is well-established, its molecular efficacy relies heavily on specific cis-acting elements within target gene promoters. The ABRE (ABA-responsive element), a specialized motif within the G-box family, serves as the primary docking site for bZIP transcription factors to mediate ABA signaling (Martignago et al., 2025; Collin et al., 2021; Hobo et al., 1999). In this study, beyond the dominant G-boxes, we identified a significant co-enrichment of 19 ABRE and 17 ARE elements across the CsBES1 family (**Figure 4**). Notably, *CsBES1-2* and *CsBES1-3* harbor the highest density of both G-box and ABRE motifs, creating a unique combinatorial regulatory landscape. This non-random clustering suggests that these specific CsBES1 members function as integrative sites, capable of simultaneously sensing light conditions (via G-boxes) and ABA-mediated stress signals (via ABREs). Such a dual-sensing architecture likely enables hemp to finely regulate BR signaling in response to complex environmental fluctuations, thereby providing a molecular basis for the enhanced alkali resistance observed in our subsequent functional assays. The prominence of these elements, alongside BOX 4 and ARE motifs involved in primary metabolism and adversity responses (Kim et al., 2024), further implies that the *CsBES1* network in hemp possesses the feature to prioritize stress adaptability over constitutive growth.

The destructive mechanism of salt-alkali stress to plants is primarily based on osmotic stress, which limits water absorption, and nutrient deficiency, which is caused by mineral element imbalance. Compared with the reversible physiological hydropenia, the negative effect of nutrient deficiency on crops, such as necrosis and chlorosis, is more complicated to solve. The previous research suggested that BR signaling is highly sensitive to nutrient deficiency. For example, N starvation can suppress BES1 accumulation and phosphorylation, thereby inhibiting its role in maintaining cellular homeostasis (Wang et al., 2019, 2023). Similarly, excessive Na^+^ competes with B for root uptake sites, leading to B deficiency that downregulates BR pathway genes and impairs root architectural plasticity. Consistent with these mechanisms, our transcriptomic data revealed a comprehensive downregulation of *CsBES1* genes under alkali stress (**Figure 5**). We propose that the downregulated genetic expression is not merely a passive response to damage but rather a strategic defense mechanism. When exposed to severe nutrient limitation and ionic toxicity, hemp may actively suppress the growth-promoting effects mediated by *CsBES1* to conserve energy and prevent elongation. This is consistent with the mechanism of action of BR signaling, which acts as a regulator balancing growth vigor and survival stress. Therefore, the observed decline in *CsBES1* expression likely represents a protective adaptive response to the dual osmotic and nutritional stresses induced by saline-alkali stress, rather than a simple loss of function.

BR has been demonstrated to be widely used as an antidote to abiotic stress resilience in plants by stabilizing cellular homeostasis, improving CO_2_ absorption, and regulating hormone levels to restore the metabolism process and defense system (Wu et al., 2019). The complicated cascade reaction between *BES1* genes and environmental factors essentially constructs an effective adversity response mechanism. For example, BZR1 could activate the *GA20ox* gene to facilitate gibberellin synthesis, thereby ensuring its function to regulate antioxidant activities and enhance photosynthetic ability and major metabolite intensities (Saini et al., 2021). Besides, previous research revealed that activated transcription factor BZR1 in the BR signaling pathway increased the ABA synthesis gene under abiotic stress conditions, comprehensively arousing the synergistic effect of ABA to regulate stomatal closure and the ROS-scavenging mechanism, along with seed dormancy, to elevate adversity tolerance. It has been shown that most of the *CsBES1* genes were suppressed under alkali stress and thus might play a significant role in adverse or negative reaction mechanisms consistent with the similar expression pattern exhibited in tomato (Su et al., 2021).

**Figure 6:**
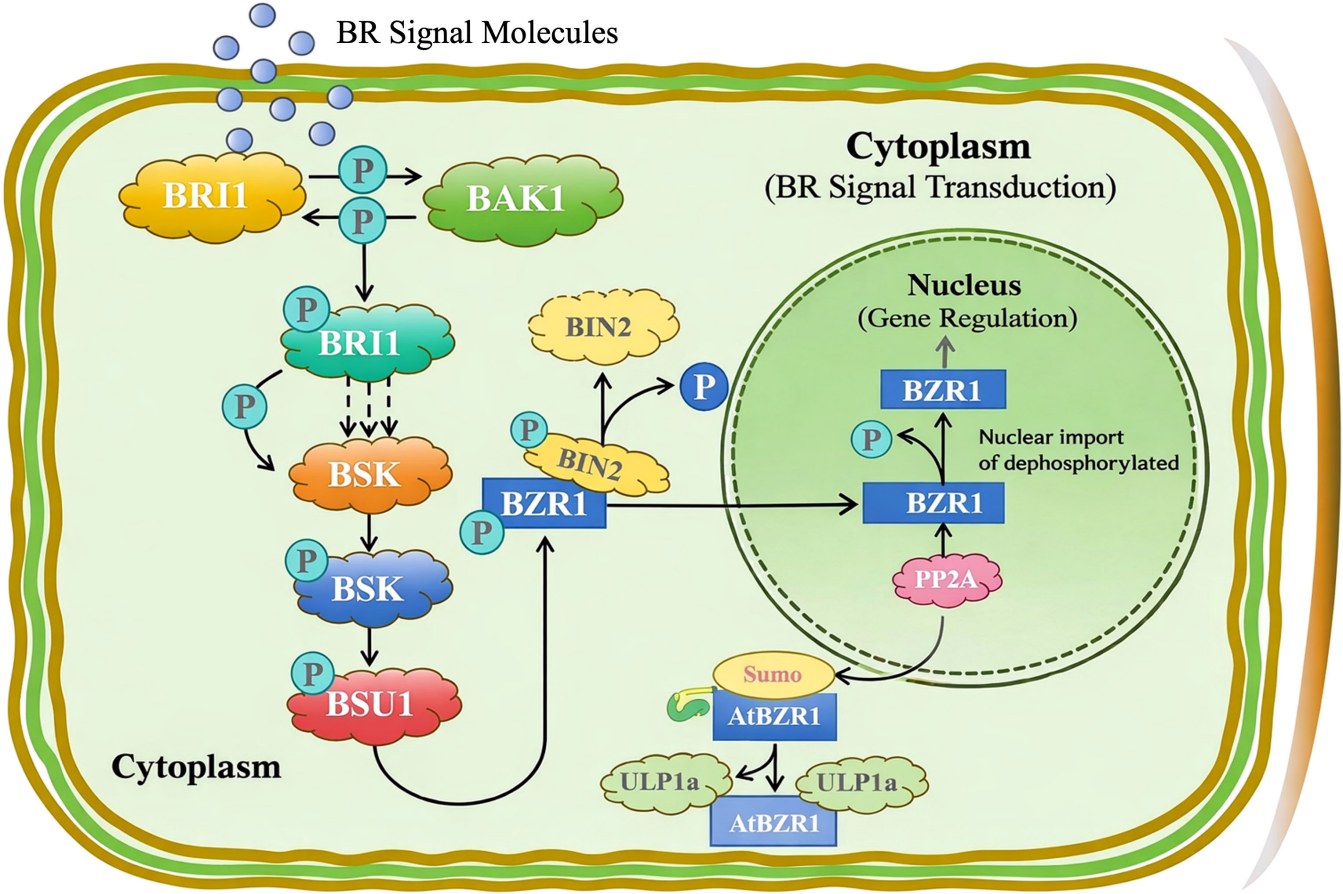
Overview of the brassinosteroid signaling cascade in the hemp plant.

Beyond localization patterns, the functional capacity of *CsBES1* genes to confer alkali tolerance was directly validated using a yeast heterologous expression system, which serves as a predictor for assessing the intrinsic stress-protective potential of target genes. Our results demonstrated that overexpression of yeast cells in *CsBES1-2* or *CsBES1-3* exhibited enhanced growth under alkali stress compared to controls harboring the empty vector (Supplementary **Supplementary Figure 3**). This finding provides causal evidence that CsBES1 proteins are sufficient to drive alkali tolerance. The ability of these hemp genes to function in yeast further underscores the evolutionary conservation of the BR-mediated stress protection mechanism, suggesting that CsBES1 activates a fundamental, ancient cellular defense program that transcends species barriers. Consequently, these genes represent prime candidates for engineering saline-alkali resilience in hemp and potentially other crops. Further, in our study, the CsBES1-2 and CsBES1-3 were found to be localized in both nuclear and cytoplasmic regions, suggesting that the BES1-like proteins in hemp are regulated by phosphorylation and dephosphorylation in a similar manner. Overall, the proposed illustrated scheme of the transduction pathway simply highlights how BR signaling is split up inside the cell: first, the signal gets picked up and passed along by kinases and phosphatases in the cytoplasm (**Figure 6**). This setup keeps BR-responsive genes under tight control, and that’s what helps plants manage growth, development, and how they handle stress.

## Conclusions

In our investigation, a comprehensive genomic characterization analysis, including chromosomal location, conserved protein sequence analysis, evolutional relationships, structural features, conserved motifs, and cis-elements, was conducted for the seven BES1 genes identified in hemp. Among these, 2 candidate genes (*CsBES1-2* and *-3*) with the most drastic expression levels and transcript patterns were further explored by subcellular localization, transactivation activity, and functional assays to explore their potential role in improving alkali stress resistance. The results clearly exhibited that the selected *CsBES1-2* and *-3* were compatible with the dephosphorylated and phosphorylated forms and possessed the potential capacity to enhance adversity tolerance. Summarily, our results lay a theoretical foundation for the functional research of the *CsBES1*-like gene family.

## Data Availability

The datasets presented in this study can be found in online repositories. The names of the repository/repositories and accession number(s) can be found below: https://www.ncbi.nlm.nih.gov/, PRJNA1163756.

## Supplementary material

Supplementary Figure 1 Subcellular localization of CsBES1-2 and CsBES1-3 through transiently expressed CsBES1-GFP fusion proteins in tobacco leaves, as perceived by the analysis of LSCM (laser scanning confocal microscopy).

Supplementary Figure 2 Transactivation activity analysis of CsBES1 proteins in yeast. The pGBKT7-CsBES1 fusion vectors were converted into the Yeast Two-Hybrid (Y2H) Gold System, cultivated on SD/-Trp medium, and protein interaction was screened through the chromogenic substrate for X-α-Gal (5-bromo-4-chloro-3-indolyl-α-D-galactopyranoside).

Supplementary Figure 3 Functional analysis of BES1 in yeast under bicarbonate stress conditions.
