# Supplementary figures and images for: Genome-wide identification and functional dissection of the BES1 family reveals key regulators of alkali stress response in hemp
Source: Front Plant Sci. 2026 Apr 30;17:1819515. doi: 10.3389/fpls.2026.1819515 (PMC13171327; doi:10.3389/fpls.2026.1819515)

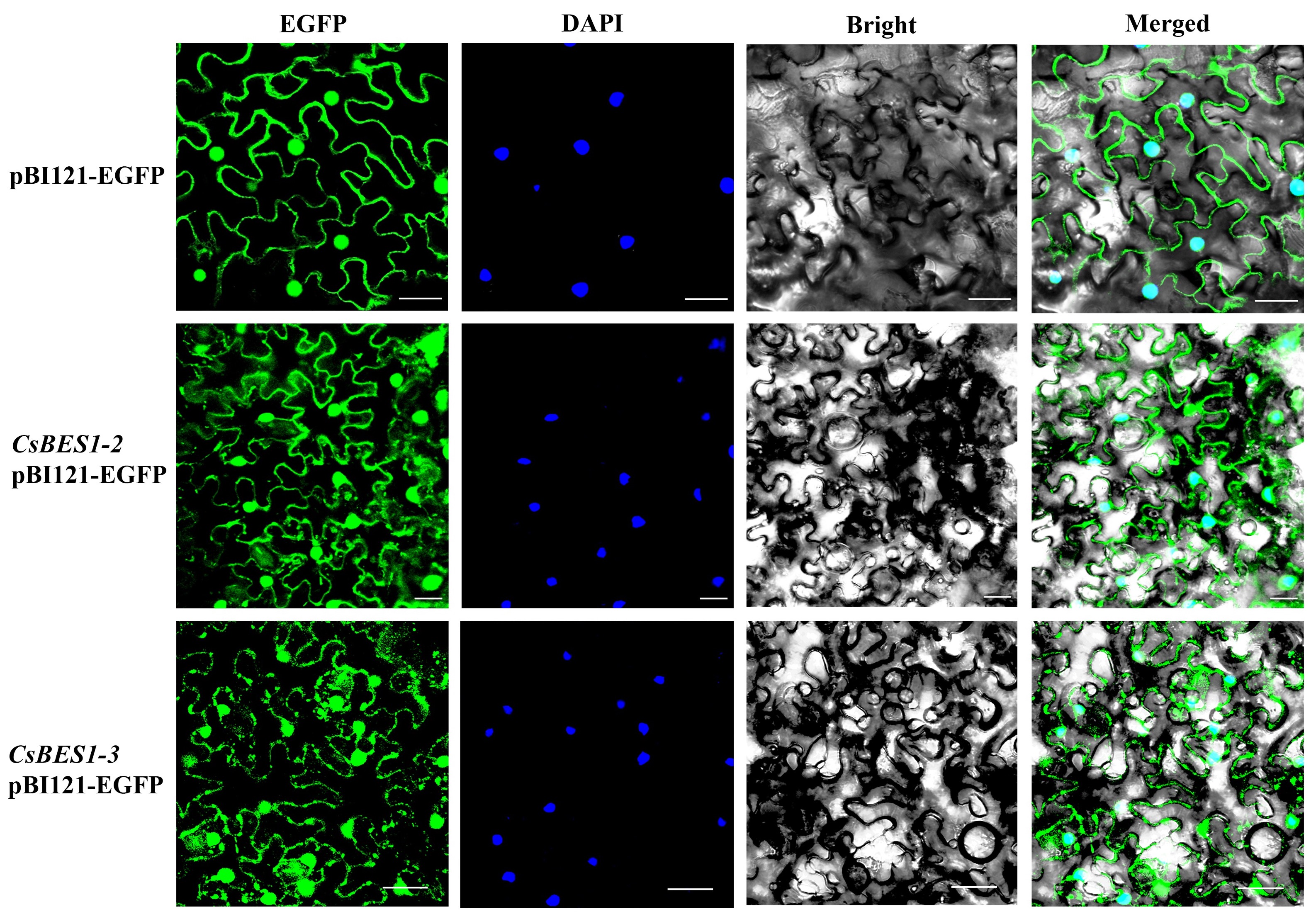

Supplement: Supplementary Figure 1 — Subcellular localization of CsBES1-2 and CsBES1-3 through transiently expressed CsBES1-GFP fusion proteins in tobacco leaves, as perceived by the analysis of LSCM (laser scanning confocal microscopy). [file Image1.jpeg]

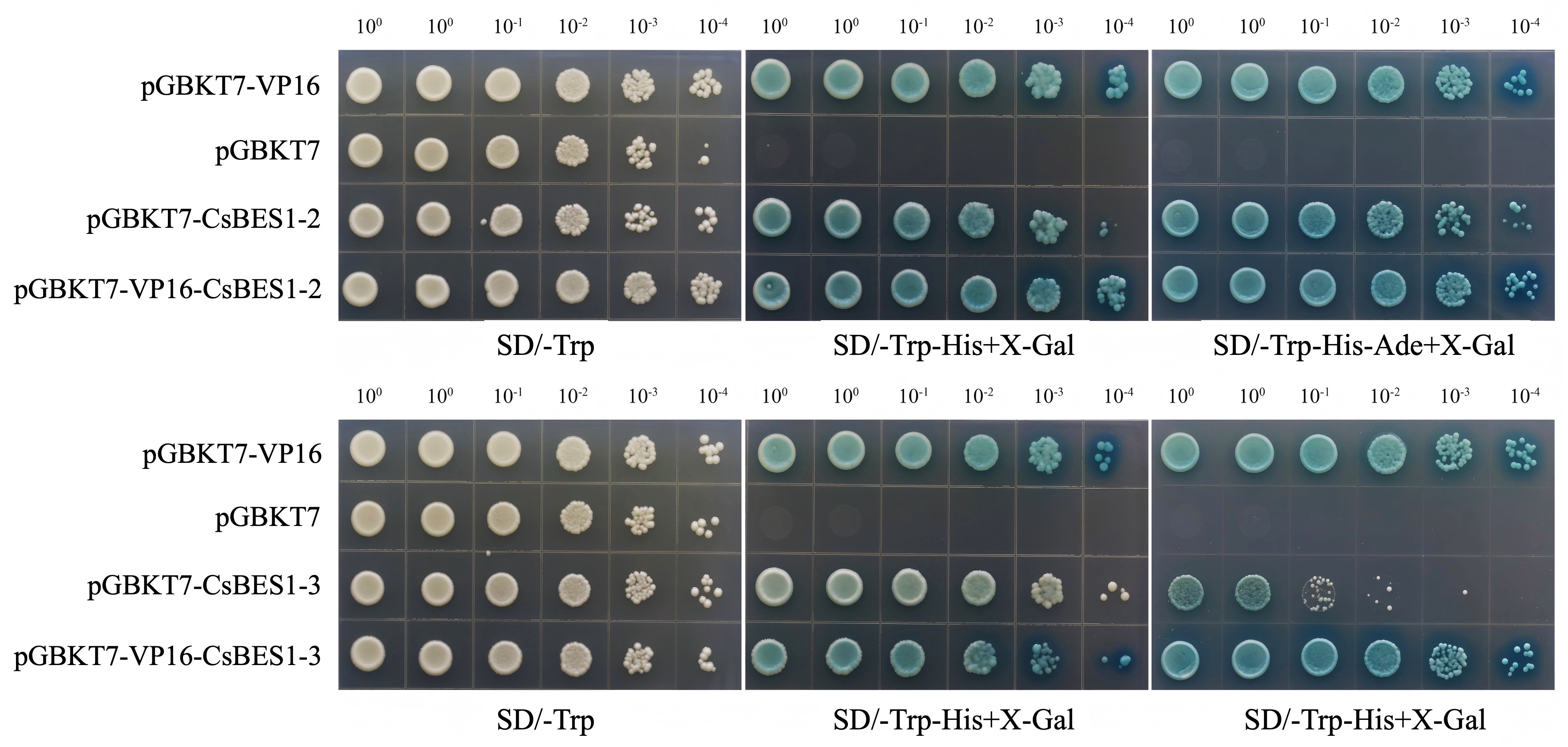

Supplement: Supplementary Figure 2 — Transactivation activity analysis of CsBES1 proteins in yeast. The pGBKT7-CsBES1 fusion vectors were converted into the Yeast Two-Hybrid (Y2H) Gold System, cultivated on SD/-Trp medium, and protein interaction was screened through the chromogenic substrate for X-α-Gal (5-bromo-4-chloro-3-indolyl-α-D-galactopyranoside). [file Image2.jpeg]

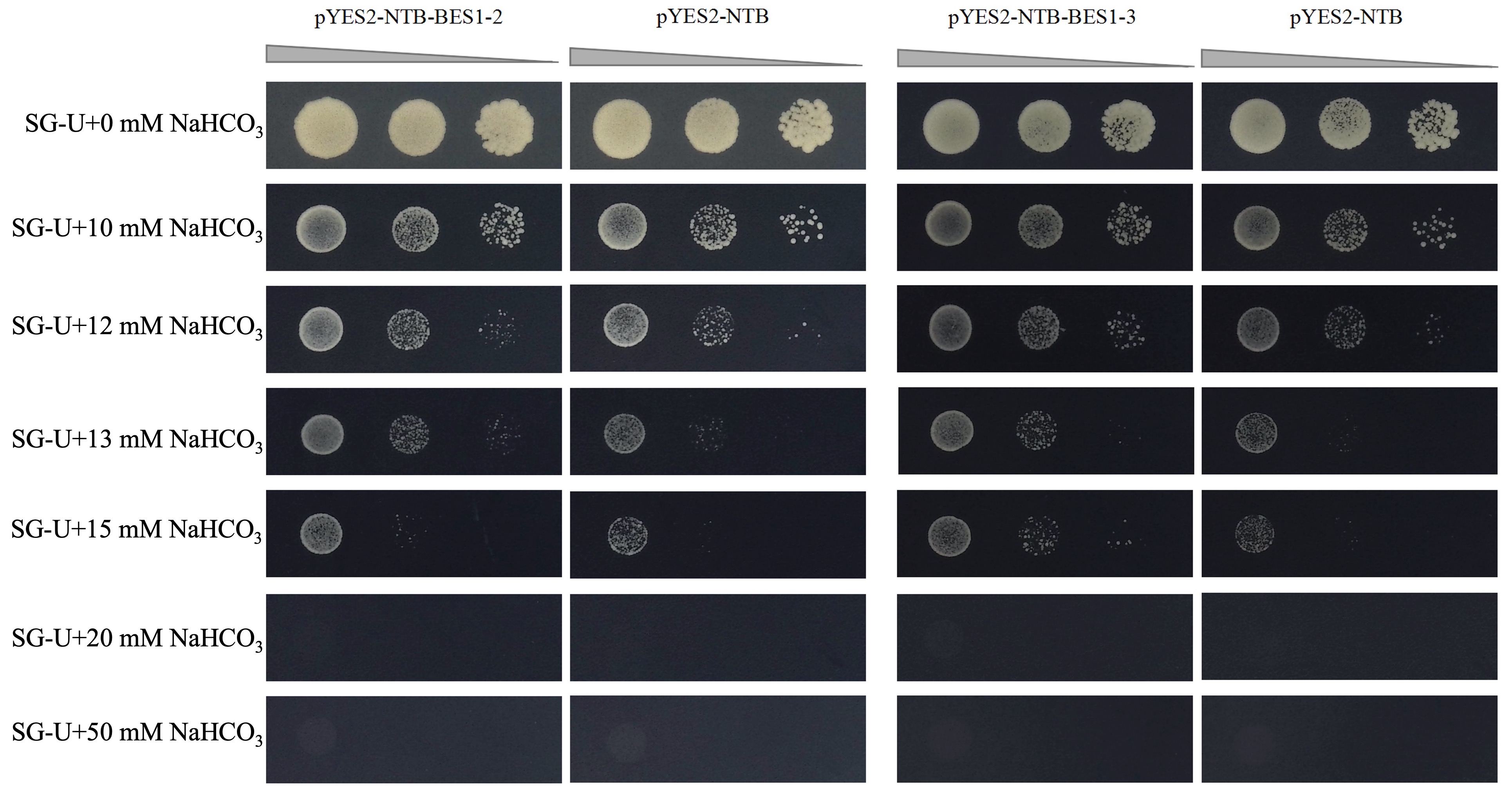

Supplement: Supplementary Figure 3 — Functional analysis of BES1 in yeast under bicarbonate stress conditions. [file Image3.jpeg]
